# Optic Nerve Sheath Meningiomas: A Narrative Review of Clinical Features, Imaging Advances, and Current Management

**DOI:** 10.3390/cancers18203405

**Published:** 2026-10-21

**Authors:** Nima Sadeghi, Kamil Laban, Peerooz Saeed

**Affiliations:** 1 Department of Ophthalmology, The Orbital Center, Amsterdam University Medical Center, 1081 HV Amsterdam, The Netherlands; 2 Department of Ophthalmology, University Medical Center Utrecht, 3584 CX Utrecht, The Netherlands

**Keywords:** optic nerve sheath, DOTATATE Scan, meningioma, radiotherapy

## Abstract

Optic nerve sheath meningioma (ONSM) is a rare orbital tumor that can pose a significant threat to visual function due to its close relationship with the optic nerve and surrounding vascular structures. Early and accurate diagnosis is essential to preserve vision and guide individualized management. The objective of this narrative review is to provide a comprehensive overview of the current evidence on the clinical presentation, diagnostic evaluation, imaging characteristics, and management of ONSM, with a particular focus on recent advances in imaging techniques and their implications for clinical decision-making. This review synthesizes current knowledge on clinical presentation, diagnostic workup, and evolving treatment strategies for ONSM, with particular emphasis on advanced imaging modalities. Magnetic resonance imaging remains the cornerstone of diagnosis, yet functional imaging, especially somatostatin receptor-–based techniques such as 68GaDOTATATE PET/CT have shown potential as a useful adjunct in the diagnostic evaluation of ONSM, especially for assessing tumor characteristics, extent, and treatment planning in selected cases. We discuss the emerging role of DOTATATE imaging in differentiating ONSM from other optic nerve and orbital pathologies, as well as its potential application in monitoring therapeutic response. We also highlight contemporary management approaches, including fractionated radiotherapy and the selective role of surgical intervention, underscoring the importance of multidisciplinary decision-making in optimizing visual outcomes. Emerging molecular insights from meningioma research may inform future therapeutic strategies for ONSM; however, clinical evidence specific to ONSM remains limited.

## Simple Summary

Optic nerve sheath meningioma is a rare tumor that grows around the nerve connecting the eye to the brain and may cause gradual, irreversible vision loss. Because its symptoms can resemble those of other eye and nerve disorders, timely and accurate diagnosis is essential. This narrative review brings together current knowledge on its symptoms, diagnosis, imaging, and treatment, with particular attention to newer scans that detect specific receptors on tumor cells. It also explains how observation, radiotherapy, and surgery are selected according to tumor growth, vision, and individual patient needs, while outlining potential targeted treatments for difficult cases. By summarizing recent advances and remaining uncertainties, this review aims to help clinicians and researchers improve diagnostic confidence, support multidisciplinary treatment decisions, preserve vision, and guide future studies of this uncommon disease.

## 1. Introduction

Meningiomas are slow-growing, benign tumors arising from the cells of the arachnoid villi. Within the orbit, they may be classified as secondary or primary [1]. Secondary orbital meningiomas account for more than 90% of orbital meningiomas and result from the intraorbital extension of nearby localizations including the sphenoid bone, clinoid bone or Sella turcica. Primary intra orbital meningiomas are considered rare and originate from the optic nerve sheath and occasionally from ectopic arachnoid cells or arachnoid rests [2].

The understanding of meningiomas has evolved considerably since Curvelier first recognized them as a distinct pathological entity in 1835 [3]. A major conceptual shift followed in 1912, when Hudson’s classification clearly separated meningiomas from gliomas [4], establishing the foundation for modern diagnostic categorization. Primary optic nerve sheath meningiomas (ONSMs) were not formally described until the late 1930s [5,6]. Early reports consistently highlighted a characteristic constellation of progressive visual decline, optic nerve pallor, and optociliary shunt vessels, a triad that was considered pathognomonic in the 20th century [1]. However, these findings are not specific to ONSM and usually do not present at the same time.

Despite increasing recognition of ONSMs and advances in diagnostic and therapeutic approaches, the available evidence remains fragmented across small retrospective cohorts, case reports, and single-centre experiences. Consequently, there is limited consolidation of contemporary evidence regarding diagnosis, treatment selection, and long-term outcomes. The primary gap addressed by this review is the absence of an up-to-date, comprehensive overview that synthesizes these disparate findings into a clinically relevant framework for practitioners and researchers. Therefore, the objective of this review is to provide a comprehensive overview of the current evidence regarding ONSM, including its epidemiology, clinical manifestations, diagnostic workup, treatment strategies, and outcomes. The review further seeks to identify areas of uncertainty and unmet clinical needs that may guide future research and improve patient care.

## 2. Materials and Methods

Given the rarity of ONSM and the resulting predominance of retrospective studies, case series, and case reports, a narrative review methodology was considered most appropriate to provide a comprehensive overview of the available evidence. A literature search was conducted in PubMed/MEDLINE for studies published up to January 2026 using combinations of the terms “optic nerve sheath meningioma”, “optic nerve meningioma”, “diagnosis”, “imaging”, “DOTATATE Scan”, “treatment”, “radiotherapy”, “surgery”, and “outcomes”. Relevant articles were identified through title and abstract screening, with additional studies retrieved through manual review of reference lists. Original studies, case series, and clinically relevant case reports focusing on ONSM were considered for inclusion, while conference abstracts without sufficient data and studies in languages other than English, German and French were excluded. Owing to the heterogeneity of study designs, outcome measures, and patient populations, a formal systematic review was not feasible. Therefore, this narrative review aims to synthesise the available evidence, provide a clinically relevant overview of current knowledge, and identify areas requiring further investigation.

## 3. Epidemiology and Etiology

Optic nerve sheath meningiomas arise from arachnoid cap cells within the meninges surrounding the optic nerve and share histopathological and molecular characteristics with intracranial meningiomas. The vast majority of cases are sporadic, and ONSMs are classified as rare tumors, representing approximately 2% of all orbital neoplasms [7,8]. Because of the extreme rarity of this disease, it is very difficult to know the incidence or prevalence of it.

Based on data from population-based analyses of orbital and optic nerve tumors reported in the Surveillance, Epidemiology, and End Results (SEER) database, the estimated incidence of ONSM is approximately 0.03–0.08 cases per 1,000,000 population. As ONSM-specific incidence rates were not directly reported in these studies, this estimate was derived from the published data and should therefore be interpreted with caution [8,9].

Unilateral presentation occurs in approximately 95% of cases [1,10]. Although bilateral ONSM cases have been documented in the literature, they remain exceptional and are frequently associated with germline *NF2* mutations [10,11,12,13,14,15,16]. The typical patient is a middle-aged adult, with ONSM presenting slightly earlier than intracranial meningiomas, which exhibit peak incidence between 45 and 55 years of age [10,11,12,14,17]. Consistent with other meningiomas, ONSMs demonstrate a marked female predominance, with women accounting for approximately 61% of cases [18], and some series reporting female-to-male ratios as high as 4:1 [1,19,20]. Hormonal influences are implicated in tumor biology, as accelerated growth has been observed during pregnancy [21]. Moreover, hormone therapy with agents such as cyproterone acetate, occasionally prescribed for menopausal symptoms, has been associated with a dose-dependent increase in intracranial meningioma risk, with risk reduction following treatment cessation [21,22].

While ONSMs occur predominantly in adults, pediatric cases are distinctly uncommon [7,8]. Fewer than 30 pediatric cases have been reported in the literature, including a case series of seven patients [16,23,24,25,26,27,28,29,30,31,32,33]. Importantly, when ONSMs arise in childhood, they are more frequently associated with neurofibromatosis type 2 (NF2), and ONSMs may represent one of the earliest clinical manifestations of this tumor-predisposition syndrome [34].

## 4. Clinical Signs and Symptoms

Symptoms of ONSM are heterogeneous and largely determined by the location of the lesion, its growth pattern, stage of disease, duration of complaints, and the initial level of visual acuity. Common symptoms include painless progressive visual loss, (transient) visual obscurations, periocular pressure and pain, and diplopia. Visual deterioration is the most common presenting symptom of ONSM, often accompanied by transient visual obscurations and periocular pain [19].

A more distinctive symptom is gaze-evoked amaurosis, in which transient ischemia of the optic nerve during eye movements leads to brief episodes of vision loss. While characteristic of ONSM, this finding is not specific and may also occur with other orbital masses such as cavernous hemangiomas. Other less frequent signs include ocular motility disturbances and strabismus [35].

On fundoscopic examination, optic disc edema and optic atrophy are the hallmark findings, sometimes appearing together, and nearly all patients show one or the other at some point [1]. Intrapapillary refractile bodies may be seen in association with longstanding optic disc edema, often at the temporal disc margin, and can precede or coincide with visual decline before disappearing as optic atrophy develops [36]. Optociliary shunts—dilated collateral vessels connecting the retinal and choroidal circulations—may also be seen, developing as a result of central retinal vein compression [37,38]. Their appearance can be delayed, and they may regress once atrophy becomes complete, suggesting that their true incidence is likely underestimated [37,39]. Although these signs provide valuable diagnostic clues, none are pathognomonic when present in isolation. Proptosis may develop as the tumor enlarges and is usually mild, slowly progressive, and often appear after visual symptoms. Lesions in the optic canal may produce significant visual loss before any noticeable external changes occur [40,41].

Slowly progressive optic neuropathies such as compressive lesions, including ONSM, and glaucoma share the common endpoint of retinal ganglion cell loss, yet their structural and functional patterns differ in clinically meaningful ways [42]. Visual field loss is present in most cases of ONSM and reflects both the site and severity of optic nerve compression, as well as the chronicity of disease. The most frequent pattern is concentric peripheral constriction, often progressing slowly over time and sometimes remaining asymptomatic until advanced [1,19]. However, a wide spectrum of defects has been described, including central, cecocentral, and paracentral scotomas, enlargement of the blind spot, and altitudinal defects; in practice, almost any pattern compatible with a compressive optic neuropathy may be observed [1,19,43]. Lesions situated near the orbital apex or with more extensive posterior involvement tend to produce more generalized constriction and diffuse depression of sensitivity across the field, whereas more anterior or segmental lesions may give rise to localized scotomas that correlate with the topography of nerve fiber bundle involvement [40,44].

By contrast, glaucomatous visual field loss typically manifests as nasal steps, paracentral and arcuate scotomas, and, in more advanced stages, wider arcuate defects and eventual tunnel vision, generally respecting the horizontal meridian and often sparing central vision until late in the disease [42] Lesions at the chiasma further add characteristic patterns, most notably bitemporal hemianopic or hemianopic-like defects when the optic chiasm is involved [40,41].

Differentiating compressive optic neuropathy and glaucoma can be challenging due to overlapping features, as both conditions contribute to optic disc changes, visual field defects, and retinal nerve fiber layer thinning [45]. Patients with normal-tension glaucoma may be especially vulnerable to diagnostic delay, as compressive optic neuropathy can mimic glaucomatous progression [46]. Therefore, atypical progression of visual loss despite adequate intraocular pressure control, asymmetric deterioration, or disproportionate loss of visual acuity and color vision should prompt consideration of optical coherence tomography (OCT) and neuroimaging to exclude compressive lesions [47].

Peripapillary RNFL analysis has emerged as a key discriminator between glaucomatous and compressive optic neuropathy and is essential in the work up of progression [47]. Temporal RNFL thinning is the most diagnostically useful parameter: patients with early to moderate primary open-angle glaucoma (POAG) generally show inferior RNFL thinning, whereas compressive optic neuropathies, including ONSM and sphenoid wing meningioma, demonstrate marked, disproportionate temporal and nasal thinning, often falling clearly outside normal limits [47,48]. Beyond structural OCT metrics, OCT angiography (OCTA) has shown that superficial macular vessel density, particularly in the peritemporal sector, is the best single parameter for discriminating between glaucomatous and compressive optic neuropathy, with compressive lesions showing significantly reduced macular perfusion across multiple sectors [48,49].

## 5. Radiographic Findings

### 5.1. Computed Tomography (CT)

On CT, ONSM typically appear isoattenuating to the optic nerve on non-contrast images and show homogeneous contrast enhancement usually with smooth tumor margins [50,51]. Best seen with contrast enhancement, enlargement of the optic nerve is seen in nearly all patients and may be focal or, more commonly, diffuse tubular; diffuse thickening is the most frequent configuration [19,50,51,52]. Other patterns such as globular, fusiform, or focal enlargement may also occur [50].

A classic CT feature with contrast is the “tram-track” sign, where the thickened, denser optic nerve sheath surrounds a central lucent nerve [50]. This sign was observed in roughly a quarter of patients [19,36]. Although suggestive of ONSM, it is not specific and may also appear in conditions such as inflammatory perineuritis [34,53]. Calcification can be better visualized on CT than MRI and is helpful for defining optic canal involvement and bone anatomy [50,52]. The presence of calcification can aid in distinguishing ONSM from optic gliomas and may also contribute to the tram track appearance [50].

### 5.2. Magnetic Resonance Imaging (MRI)

MRI has become the preferred modality for diagnosing ONSM, due to its contrast and fat suppression techniques, making it superior to CT in assessing soft tissue and evaluating involvement of the optic canal and intracranial extension [34,52,54,55].

Gadolinium enhancement highlights sheath thickening, typical for meningiomas, producing the classic “tram track” sign on axial images where the enhancing tumor flanks the non-enhancing central optic nerve [56]. Careful examination of the orbital apex and optic canals can be important for identifying small intracanalicular tumors [54].

**Figure 1:**
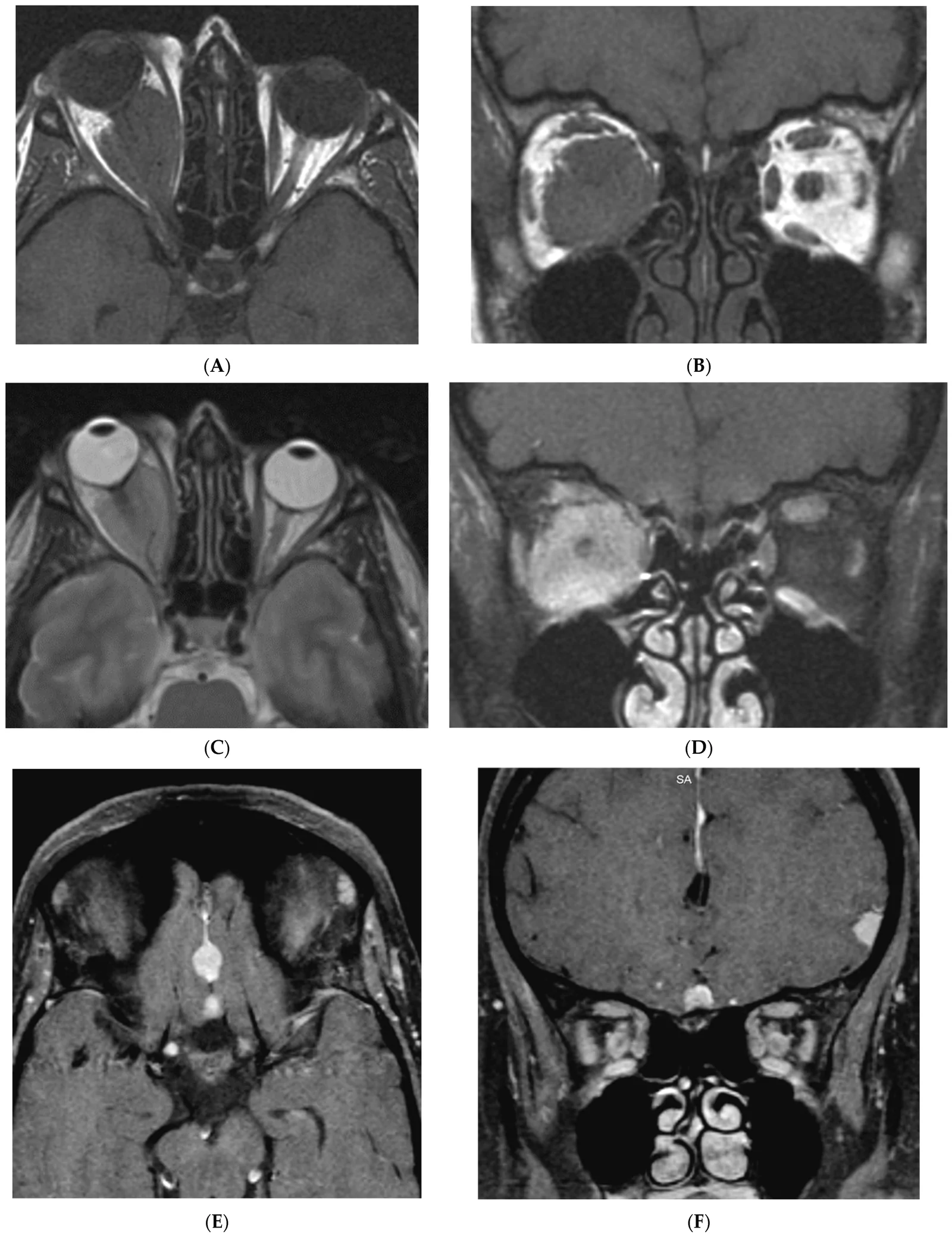
(**A**) Axial T1-weighted MRI demonstrating a globular ONSM involving the right optic nerve. The lesion shows longitudinal thickening along the course of the optic nerve, consistent with the globular growth pattern of ONSM. (**B**) Coronal T1-weighted MRI demonstrating the same ONSM, with circumferential sheath thickening around the optic nerve. (**C**) Post-gadolinium, axial T2-weighted MRI demonstrating a tubular ONSM of the right eye, with circumferential thickening of the optic nerve sheath along the course of the optic nerve. (**D**) Coronal view of the same lesion with circumferential thickening and compression of the right optic nerve. (**E**) MRI with fat suppression in axial plane after contrast injection showing bilateral ONSM. (**F**) Coronal plane showing thickened optic nerve sheaths bilaterally with associated multiple intracranial meningiomas in the same patient.

Optic canal involvement is a common feature occurring in ONSM [1,19]. Intracranial extension occurs in a minority of cases, and the morphologic classification proposed by Saeed et al. provides a useful framework for characterizing these tumors [19], as shown in Figure 1. The tubular-diffuse pattern is the most common, marked by subtle longitudinal nerve widening and frequent calcification with tram tracking. Less common tubular variants include apical expansion, often associated with proximal intracranial involvement and anterior expansion toward the globe. The globular, exophytic pattern represents the second most frequent morphology, distinguished by growth beyond the dural sheath, irregular margins, and pronounced proptosis. Fusiform enlargement and focal, localized thickening of the optic nerve constitute the remaining, less prevalent presentations [19].

On T1-weighted images, ONSMs typically appear isointense to hypointense relative to the optic nerve or brain tissue [20,57]. The tumors generally have smooth, well-defined borders and demonstrate homogeneous enhancement after gadolinium administration. On T2-weighted images, they are often isointense to hyperintense compared to the optic nerve [57]. This T2 hyperintensity is helpful for differentiation because it contrasts with the expected T2 hypointensity or isointensity of optic nerve gliomas [50]. Additionally, ONSM often show restricted diffusion due to their hypercellular nature, which can further distinguish them from inflammatory or demyelinating processes [56].

### 5.3. Somatostatin Receptor Scintigraphy

ONSMs show a characteristic protein expression profile that supports both routine diagnosis and functional imaging. They typically express vimentin and epithelial membrane antigen (EMA), with frequent progesterone receptor (PR) positivity, a pattern shared with other benign meningiomas. Somatostatin receptor subtype 2A (SSTR2A) is strongly and diffusely expressed in nearly all meningiomas [58], including skull-base and meningothelial subtypes that correspond to the most common ONSMs phenotype. Large tissue-microarray studies have shown SSTR2A immunostaining to be more sensitive and at least as specific as EMA and PR for identifying meningioma, with essentially 100% of tested meningiomas showing strong membranous/cytoplasmic SSTR2A expression, while most potential mimickers (schwannoma, MPNST, metastases) are negative or only weakly positive [59].

**Figure 2:**
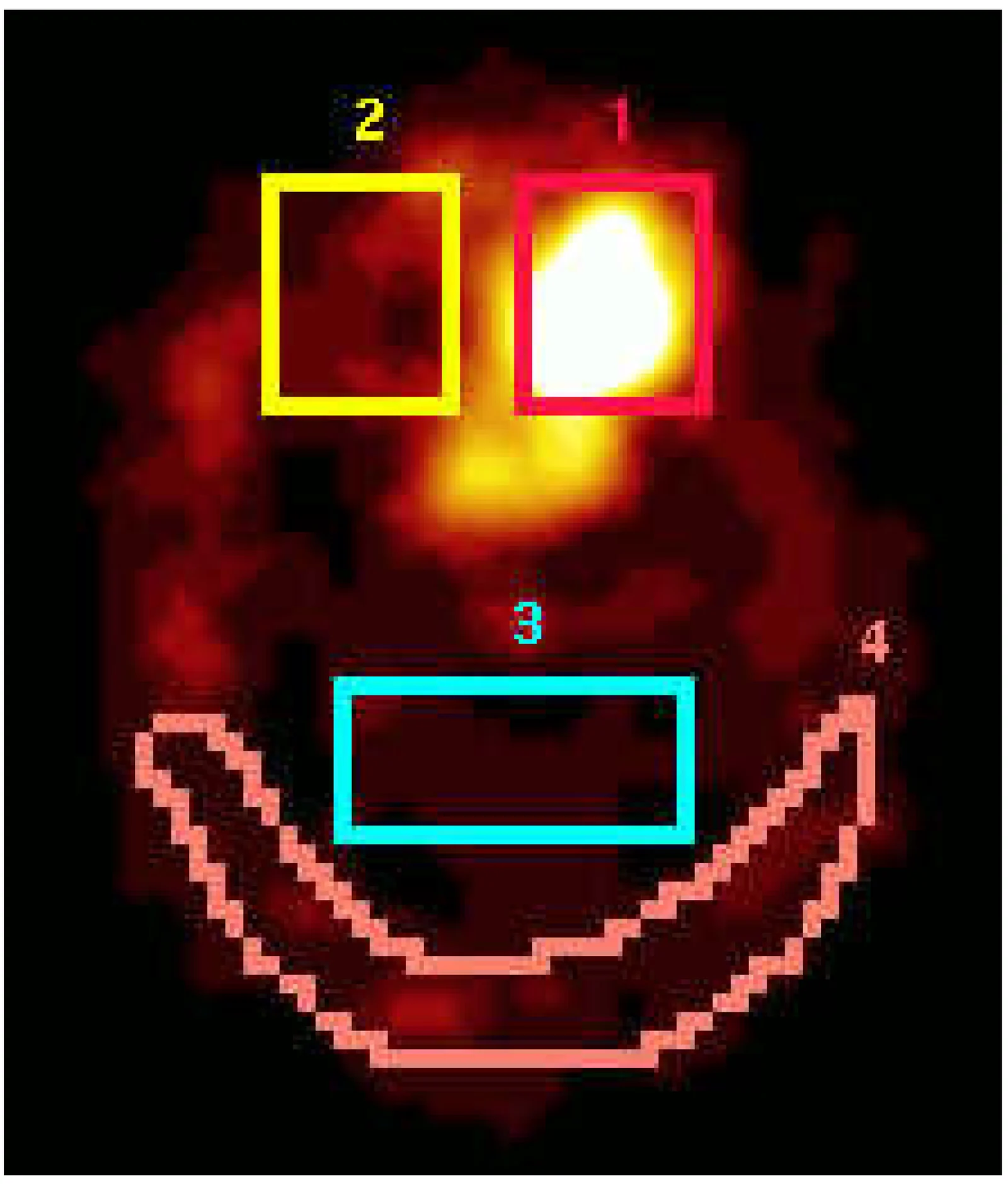
Scintigraphy of ONSMs. Somatostatin-receptor scintigraphy demonstrating focal radiotracer uptake along the optic nerve sheath, consistent with somatostatin receptor positive ONSM.

As in all meningiomas, ONSMs express high levels of somatostatin receptors (particularly the sst2A subtype) [58], enabling visualization with radiolabeled somatostatin analogues [58,60,61]. At a molecular level, these tracers bind selectively to somatostatin receptors on meningioma cells, so that areas of dense receptor expression accumulate radiotracer and can be detected as focal “hot spots” on functional imaging, as demonstrated in Figure 2. SPECT/CT using 99mTc-HYNIC-TOC is highly sensitive and specific for ONSM, providing detailed images with excellent spatial resolution [62]. This technique improves diagnostic confidence by confirming and localizing somatostatin receptor-positive lesions, while distinguishing ONSM from optic glioma or inflammatory perineuritis, which typically show minimal uptake.

It is especially useful when MRI findings are inconclusive or when biopsy is not feasible, offering a non-invasive, highly specific tool that can obviate the need for tissue diagnosis and can also assist in radiotherapy planning and treatment response assessment [62].

### 5.4. Ga-68 DOTA-TATE PET-CT

Ga-68 DOTA-TATE PET-CT combines a somatostatin analog with a Gallium-68 tracer to visualize somatostatin receptor uptake. Although widely used for neuroendocrine tumors [63], it is also effective in detecting meningiomas due to their somatostatin receptor overexpression [60,64,65]. Somatostatin receptor PET with Ga-68–labelled analogues (DOTATATE/DOTATOC) has emerged as a highly accurate adjunct for diagnosing ONSMs and other anterior optic pathway meningiomas [64,65]. Many ONSMs strongly express sst2A receptors, leading to intense, sheath-based tracer uptake that contrasts sharply with the low or absent uptake seen in key mimickers such as optic glioma, optic neuritis/perineuritis, lymphoma, or leukemic infiltration. meningiomas can be distinguished from non-meningiomatous lesions by markedly higher SUVmax values of Ga-68-DOTATATE PET/CT, with 100% sensitivity and 100% specificity [60].

**Figure 3:**
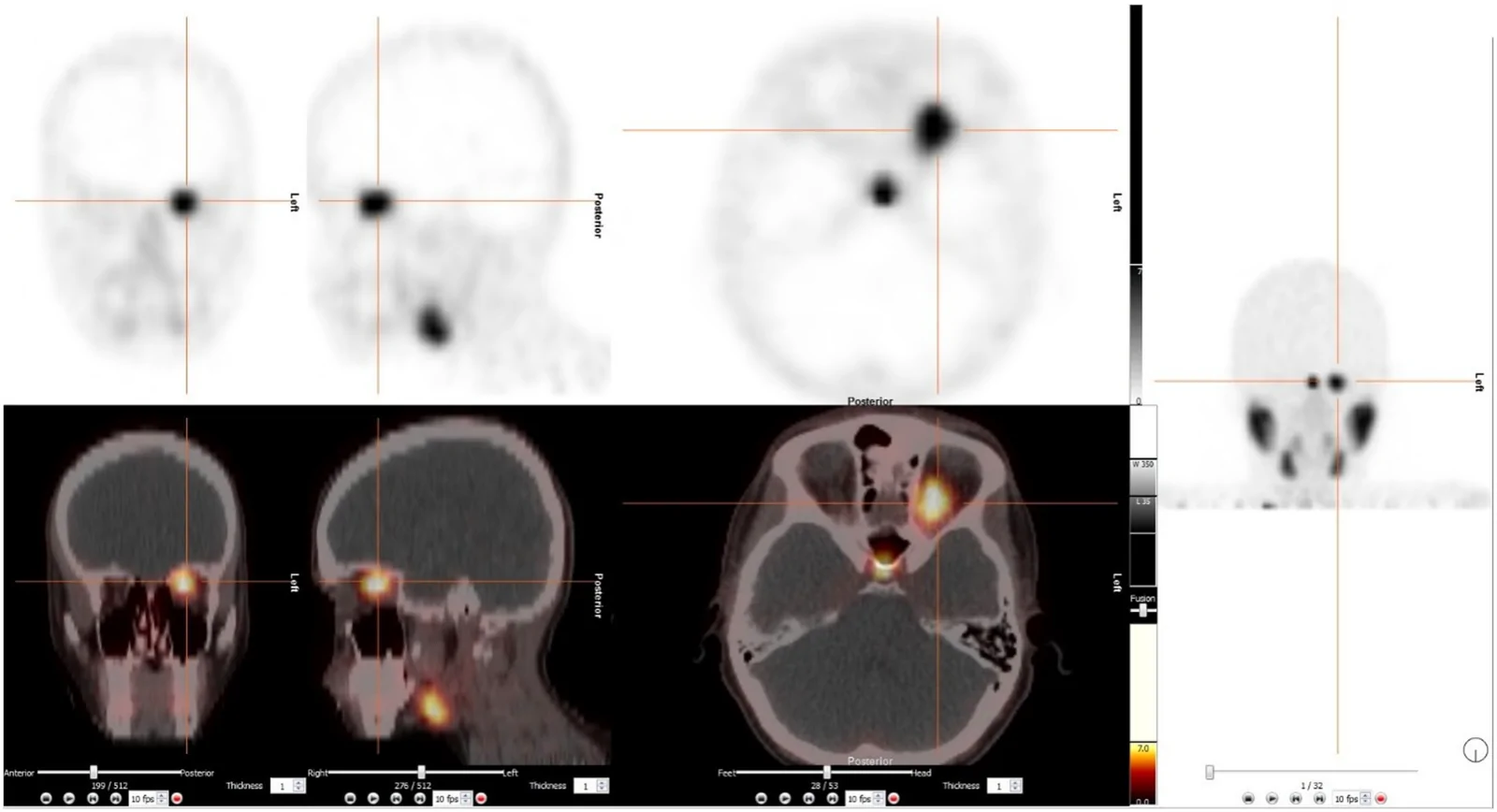
^68^Ga-DOTATATE PET-CT in coronal, axial and sagittal plane demonstrating enhancement of the left optic nerve-sheath complex with intense radiotracer uptake in the left ONSM.

Compared to Somatostatin Receptor Scintigraphy using In-111-pentetreotide (OctreoScan), Ga-68 DOTA-TATE PET-CT offers several advantages. PET-CT provides superior spatial resolution, higher target-to-background ratio, shorter acquisition times, and lower radiation doses [66]. The hybrid PET-CT fusion enables precise anatomical localization of receptor-positive lesions, as is pictured in Figure 3, which is particularly valuable in the confined orbital space where exact delineation of optic nerve sheath involvement is critical^52^. While SRS remains useful in centers without PET capability, Ga-68 DOTA-TATE PET-CT is now preferred when available due to its improved sensitivity, specificity, and diagnostic confidence in differentiating ONSM from its mimickers [66].

## 6. Histopathology

### 6.1. Genetics

Molecular characterisation of ONSM remains limited because of the rarity of the disease and the infrequent availability of tumor tissue for genetic analysis.

The majority of ONSMs harbor pathogenic variants in genes such as TRAF7, KLF4, AKT1, or SMO, identifiable through targeted next-generation sequencing panels designed for meningioma associated genes [67,68].

Chromosome 22q alterations occur in a subset of ONSMs through an alteration of the NF2 gene encoding merlin, a cytoskeletal adaptor protein that regulates contact dependent growth inhibition and multiple intracellular signaling pathways [68,69]. Typically, alterations occur through monosomy or segmental loss of chromosome 22q coupled with a second hit point mutation in NF2 [69]. This genetic pattern is particularly prevalent in NF2-associated ONSM but is also observed in sporadic cases. The loss of merlin function leads to unchecked proliferation of meningothelial cap cells along the optic nerve sheath, mirroring the biology of NF2-driven cranial meningiomas [70]. From a diagnostic standpoint, cytogenetic assays assessing 22q status combined with targeted sequencing of NF2 constitute the core genetic tests when NF2-related disease is suspected, particularly in younger patients, bilateral tumors, or those with additional features suggestive of neurofibromatosis type 2 [71,72].

Accordingly, comprehensive genetic evaluation of ONSM increasingly relies on an integrated approach combining chromosome 22q analysis with targeted sequencing of NF2 and established non NF2 driver genes to clarify tumor pathogenesis, identify syndromic associations, and inform biologic classification and potential therapeutic strategies. Nevertheless, it is important to recognise that several molecular insights currently applied to ONSM are extrapolated from studies of intracranial meningiomas owing to the limited availability of ONSM-specific genomic data. Further dedicated molecular studies are therefore needed to better define the genetic landscape of ONSM and determine the extent to which it differs from meningiomas at other anatomical sites.

### 6.2. Grading

ONSMs are overwhelmingly WHO grade I tumors [19,73], and higher-grade lesions are exceptionally rarely reported in the literature in this location [74]. Only isolated reports describe atypical or anaplastic ONSMs, and many of these remain uncertain because histopathologic confirmation is seldom obtained, or when obtained, it is also WHO grade I [74,75,76,77]. Biopsy carries a high risk of vision loss and is therefore almost never performed [20]. As a result, the true incidence of higher-grade ONSM is unknown, but based on available clinical and radiologic experience, ONSMs should be considered predominantly benign, grade I tumors, with the caveat that grading cannot be definitively established without tissue. Histopathologic prognostic factors in ONSM are limited by the rarity of biopsy but are largely extrapolated from intracranial meningioma data. WHO grade is the most important determinant, with grade I tumors demonstrating excellent prognosis and slow growth, while the rare grade II or III lesions are associated with worse outcomes and higher recurrence risk [19,73]. The Ki-67 proliferation index is also prognostically significant: values > 4% correlate with worse overall survival and disease-free survival, whereas low Ki-67 is typical in benign ONSM [19,78].

### 6.3. Role of Biopsy

Before the advances in imaging, biopsy with histopathological confirmation was required to diagnose ONSM. With modern imaging diagnosis can now be made confidently based on clinical and radiologic findings alone [79]. Biopsy is reserved for atypical cases where imaging is inconclusive, as the procedure carries a significant risk of damaging the optic nerve or adjacent structures. The potential benefit must therefore clearly outweigh the risk.

## 7. Treatment

Historically, surgical removal has been the cornerstone of therapy for decades [1,80,81,82].

Today, an individualized treatment approach is applied based on patient demographics, clinical presentation, and tumor location. Observation is recommended for asymptomatic patients or those with minimal visual deficits and no radiographic progression [19,81,82,83]. Fractionated radiotherapy remains the primary treatment for symptomatic ONSM, achieving tumor control and visual preservation in the majority of cases. Alternative hypofractionated regimens, such as 25 Gy in 5 fractions, have demonstrated comparable safety and efficacy for local control and visual function preservation [52,84,85,86]. Surgery is generally reserved for cases with severe proptosis, blind painful eyes, or tumors extending beyond the orbit with intracranial involvement [1,80,87,88].

### 7.1. Observation

Because ONSMs rarely affect mortality [1], management is centered on preserving visual function. Therefore, clinicians must balance the potential harms of intervention against the risk of progressive, irreversible vision loss under observation. The standard clinical approach is to observe patients with neuroimaging and neuro-ophthalmologic tests, including visual acuity and visual field [19,82,83,89]. However, this conservative strategy carries the countervailing risk that subtle but true progression may go unrecognized until damage to retinal ganglion cells and the optic nerve becomes functionally significant and only partly reversible. Treatment is generally initiated once objective visual deterioration or radiologic progression becomes evident, reflecting a considered trade-off between the risks of intervention and the risk of vision loss if the tumor is left untreated.

We note that the majority of untreated patients eventually experience progressive visual decline [1,52,80], and only a minority maintain vision beyond five years [19,83]. Smaller observational cohorts have reported occasional long-term stability, including rare cases with decades of preserved vision, but these remain exceptions rather than the rule [81,83,90,91]. Patients managed with observation alone may show mild decline or remain stable only when vision is already poor at presentation [78].

### 7.2. Radiotherapy

Radiotherapy has become an established vision-preserving treatment for ONSM, evolving from early radium and conventional external beam techniques to modern conformal, stereotactic and proton-based approaches [52,92,93,94,95]. Its widespread adoption was delayed due to concerns about radiation-induced damage to the optic apparatus and surrounding structures [96]. Since the 1980s, advances in planning, immobilization and dose delivery have enabled precise targeting with steep dose gradients, establishing radiotherapy as firstline treatment in symptomatic eyes with useful vision [52,88,94,97,98,99,100].

Modern fractionated external beam techniques, including three-dimensional conformal radiotherapy, fractionated stereotactic radiotherapy (FSRT), conventionally fractionated stereotactic radiotherapy (CF-SRT), intensity-modulated radiotherapy (IMRT) and conformal fractionated radiotherapy (FCRT), typically deliver 45–55 Gy in daily fractions of 1.7–2.0 Gy, most commonly 50–54 Gy in 25–30 fractions [93,101,102]. These regimens maximize tumor control while respecting optic nerve tolerance, using stereotactic localization, rigid immobilization and image guidance to spare adjacent structures [92,93,103]. Radiographic local control approaches 100% at 3–5 years, with most tumors remaining stable or showing modest reduction on imaging [86,104,105,106].

IMRT achieves steep dose gradients through beam modulation across multiple fields, improving conformity compared with conventional three-dimensional conformal radiotherapy (3D-CRT) [102,107]. Clinical series report local control rates above 90%, with visual stabilization or improvement in many patients [102,108]. It is particularly useful for tumors extending to the orbital apex or intracranially, where surgical morbidity is high. Standard dosing is 50–54 Gy in 1.8–2 Gy fractions, balancing tumor control with the risk of radiation-induced optic neuropathy [107,108]. Overall, IMRT provides effective organ-preserving treatment with favorable visual outcomes.

Functional outcomes show that fractionated conformal and stereotactic radiotherapy maintain or improve visual acuity and fields in most treated eyes with residual vision [93,97,104,109,110]. Approximately two-thirds to over 90% of eyes preserve or gain acuity, particularly when treatment is initiated before severe or prolonged vision loss [85,99,111]. Eyes already blind rarely recover vision, though tumor control remains durable [93,112]. Shorter symptom duration and optic disc swelling (rather than atrophy) are associated with better outcomes, supporting early intervention [99,103,113].

Stereotactic techniques such as FSRT and CF-SRT are widely used because they provide highly conformal dose distributions around the optic nerve sheath while sparing adjacent tissues [86,101,103]. They are suited to various tumor growth patterns and can be tailored to lesion extent [94,102].

Modern stereotactic and highly conformal techniques are generally preferred because they permit better dose conformity and limitation of radiation exposure to adjacent critical structures, although direct comparative evidence between techniques remains limited [98,101,103,110].

Stereotactic radiosurgery and hypofractionated approaches (including Gamma Knife, CyberKnife and multisession radiosurgery) are used in selected patients [94,100,114]. These deliver large doses in one or few fractions with excellent local control in carefully chosen cases but require meticulous planning due to proximity to the optic apparatus; fractionated regimens are preferred when the tumor encases the nerve [100,115]. Visual outcomes are variable and depend on baseline function and dose, with risks including delayed radiation-induced optic neuropathy, retinopathy, cranial neuropathies and rare vascular events [105,106,116]. Fractionated radiotherapy (50.4–54 Gy in 27–28 fractions) achieves comparable tumor control with lower toxicity due to reduced per-fraction dose s [110,112,117]. Accordingly, fractionated radiotherapy is generally preferred over single fraction radiosurgery when vision preservation is critical [103,110]. Careful adherence to dose constraints and long-term follow-up remain essential. Proton beam therapy and other particle techniques offer sharp dose fall-off with high conformity and good outcomes, but availability is limited to specialized centers [105,106,118].

Peptide receptor radionuclide therapy (PRRT) is an emerging option for advanced or recurrent meningiomas expressing somatostatin receptor subtype 2 (SSTR2) [119]. It uses radiolabeled somatostatin analogues such as [^177^Lu]-DOTATATE to deliver targeted radiation [66]. Meta analyses show disease control in about 67% of patients with favorable tolerability [120]. ONSM typically shows strong SSTR2 expression, enabling both potential treatment and differentiation from optic gliomas using [^68^Ga]-DOTATATE PET/CT [61]. Although early results for intracranial meningiomas are promising [121], specific data for ONSM remain limited.

A recent case report described successful [^177^Lu]-DOTATATE therapy in a 23-year-old woman with refractory ONSM, with marked reduction in proptosis after three cycles. It also reported the first fibroblast activation protein inhibitor uptake in ONSM on [^99m^Tc]-FAPI scintigraphy, suggesting potential for dual SSTR and FAP-targeted approaches [65]. These findings support further investigation of PRRT as a salvage option for refractory disease.

Radiation-related complications include retinopathy, dry eye, cataract, radiation-induced optic neuropathy and, less commonly, hypothalamic–pituitary dysfunction or temporal lobe changes [96,99,105,106]. With modern fractionated stereotactic techniques and strict dose constraints, these are uncommon and usually mild; most series report high tumor control and visual preservation with few significant adverse events, typically in patients receiving higher doses or with preexisting damage [98,99,101,109]. Nonetheless, potential severe morbidity should be discussed during counselling, especially when doses approach tolerance thresholds [95,96,110].

### 7.3. Surgery

Surgery for ONSM is primarily reserved for blind, painful eyes with severe proptosis or for cases with intracranial extension and radiotherapy failure [19,81,122,123]. In eyes with useful vision, circumferential optic nerve sheath resection is generally avoided due to the high risk of severe visual loss from devascularization and mechanical injury [1,19,80,124]. Because ONSMs share their blood supply with the pia mater, resection often disrupts pial vessels, causing ischemic optic nerve damage. In sighted eyes, intraorbital surgery is therefore limited to diagnostic biopsy, optic canal decompression, or carefully selected debulking of small anterior lesions, with strict preservation of vascular supply and axonal integrity [19,80,125,126]. In contrast, in blind eyes or after radiotherapy failure, surgery may be performed to achieve local tumor control, reduce proptosis and relieve pain, using approaches such as en-bloc intraorbital optic nerve resection, exenteration or combined cranio-orbital procedures [19,80,126,127].

Transcranial and combined cranio-orbital approaches remain standard for large intraorbital ONSM with orbital apex or intracranial extension. Typically performed via a pterional craniotomy, these involve intra- and extradural exposure, optic canal unroofing, and subtotal or in blind eyes, gross total tumor removal, with resection of intracranial extension toward the chiasm when required [19,81,126,127,128,129]. The goals are optic nerve and canal decompression, reduction in orbital and anterior cranial fossa mass effect, and interruption of tumor spread. Lateral orbitotomy, with or without rim removal, is used for anterior or lateral lesions to allow biopsy, debulking, decompression or exenteration, particularly in patients with poor vision or disfiguring proptosis [19,80,126,127,129].

Endoscopic endonasal and transnasal approaches have emerged as minimally invasive alternatives, especially for inferomedial or canalicular tumors [123,130,131,132,133,134]. These provide access to the medial orbit and optic canal for bony decompression, canal opening, and, in selected cases, limited sheath opening and tumor removal. The aim is to relieve compression while minimizing manipulation of the nerve and its vascular supply [123,131,132,133,134]. In blind eyes, endoscopic decompression may also reduce proptosis and limit progression toward the chiasm, either as an alternative or adjunct to transcranial surgery [123,130,131,132,133,134].

Surgery is increasingly used as part of a multimodal strategy rather than a curative or vision-restoring treatment alone. It is largely restricted to eyes with severely impaired vision, patients with significant proptosis, or tumors with intracranial extension threatening the chiasm or contralateral optic nerve, often combined with radiotherapy or observation depending on individual risk [1,19,80,81,82,122,123,125,128,130,131,132,133,134,135].

Prognosis after surgery is generally guarded, with highly variable visual outcomes. Historical series show a high risk of postoperative visual decline or blindness when tumors encasing the optic nerve are dissected, supporting restriction of surgery to blind or nearly blind eyes or selected exophytic/globular lesions causing disfiguring proptosis [136]. In carefully selected intraorbital exophytic or globular ONSM, about one-third of patients improve and roughly half maintain stable vision after debulking [137], indicating that conservative decompression may be feasible when preoperative vision is relatively preserved.

Surgical resection carries substantial risks, particularly permanent vision loss due to disruption of the shared pial blood supply [1,80,88]. In eyes with useful vision, surgery often results in marked visual deterioration or blindness [1,19,80]. Earlier decompression techniques were also associated with tumor seeding into orbital tissues [17]. Additional complications include proptosis, intracranial injury, cerebrospinal fluid leakage and rare extension to the optic chiasm [1,17,80,87,124,126,138]. Poorer visual outcomes correlate with longer symptom duration, longer follow-up and tumor location within the optic canal^58^. Given these risks, surgery in eyes with preserved vision is generally avoided in favor of radiotherapy, which offers superior visual preservation.

### 7.4. Targeted Therapy

Several novel targeted and immunotherapeutic approaches are under investigation for recurrent and high grade meningiomas [139,140], with potential implications for ONSM refractory to conventional treatment. Antiangiogenic therapy with bevacizumab has demonstrated promising activity in recurrent meningiomas, achieving 6-month progression-free survival rates of 93%, 85%, and 51% for WHO grade I, II, and III tumors, respectively [141]. Bevacizumab appears particularly promising because meningiomas are often highly vascular and may depend on VEGF-mediated angiogenesis; in addition, VEGF blockade may reduce peritumoral edema, which can contribute to symptom relief and improved clinical stability. Combination regimens with everolimus (mTOR inhibitor) have shown similar efficacy with prolonged disease control [142]. The CDK4/6 inhibitor abemaciclib has demonstrated activity in genomically driven trials, with 58% of patients with NF2-mutant or CDK-pathway-altered high-grade meningiomas achieving 6-month progression free survival [143]. Immune checkpoint inhibitors, particularly pembrolizumab and nivolumab, have shown durable responses in select patients with recurrent WHO grade II/III meningiomas, especially those with high mutational burden or mismatch repair deficiency [144,145]. Hormonal approaches have also been explored: antiprogestins such as mifepristone (RU-486) achieved disease stabilization in select cases, particularly women with multiple progesterone receptor-positive meningiomas [146,147], though a large phase III trial failed to demonstrate benefit in unselected patients [148]. Similarly, the somatostatin analog octreotide has shown modest efficacy in meningiomas, particularly skull base tumors, with 6-month progression-free survival reaching more than 90%, supporting its potential role as adjunctive therapy [149,150]. Among these approaches, bevacizumab currently appears especially promising because it has a consistent disease control signal and may also improve tumor-associated edema, although the evidence remains indirect for ONSM. While these systemic approaches have not been specifically studied in ONSM, the shared molecular characteristics with intracranial meningiomas suggest potential applicability for recurrent or radiation-refractory ONSM, warranting further investigation in this rare tumor subtype.

### 7.5. Discussion

Although several genetic alterations have been identified in meningiomas, it is important to distinguish between findings demonstrated specifically in ONSMs and those inferred from studies of intracranial meningiomas. Due to the limited availability of ONSM tissue and the rarity of the disease, dedicated molecular data remain scarce. As a result, many proposed genetic associations in ONSM are extrapolated from larger intracranial meningioma cohorts and may not fully reflect the molecular characteristics of ONSM.

NF2-associated ONSM typically presents at a younger age and is more often bilateral or part of a multifocal meningioma phenotype, whereas sporadic ONSM is usually unilateral and isolated. Although individual lesions have similar imaging features, suspected NF2-associated disease warrants assessment for bilateral involvement and additional intracranial tumors. Treatment principles are similar, but multifocal disease may complicate surveillance and planning. Visual deterioration may be more aggressive in pediatric and NF2-associated cases, although direct comparative prognostic data remain limited.

It is also important to distinguish between therapeutic approaches supported by established clinical evidence and those that remain investigational. Fractionated radiotherapy is currently considered the standard treatment for progressive or symptomatic ONSM and has demonstrated excellent long-term tumor control with preservation or improvement of visual function in the majority of patients. Recent systematic review data reported high tumor control rates supporting the central role of radiotherapy in contemporary ONSM management [98]. In contrast, emerging strategies, including molecularly targeted therapies and other novel treatment approaches, are supported primarily by limited preclinical evidence, small clinical series, or extrapolation from intracranial meningioma populations. While these approaches may hold promise for future management, their efficacy and safety in ONSM remain incompletely characterized, and further prospective investigation is required before their routine clinical implementation can be recommended.

### 7.6. Follow-Up

Follow-up aims to detect progressive visual decline under observation (triggering consideration of radiotherapy), to confirm radiographic control after surgery or radiotherapy, and to identify late radiation related complications such as retinopathy or pituitary dysfunction that may require ophthalmic or endocrine management. Given the chronic, slow-growing nature of ONSM and the aim of preserving vision over decades, structured long-term follow-up is essential. Standard practice is to monitor patients with regular neuro-ophthalmologic examinations with periodic orbital and cranial MRI to assess tumor size, morphology and extension [94].

## 8. Prognosis

ONSMs typically follow an indolent but slowly progressive course, with visual loss as the main source of morbidity [1]. Untreated, many patients experience gradual deterioration of visual acuity and visual fields over years, although a subset show long-term stability [19,82,83]. Modern fractionated stereotactic radiotherapy substantially improves the functional outlook, with pooled contemporary series and meta-analytic data suggesting that almost all patients achieve durable local tumor control and that roughly 80–90% have stable or improved visual acuity and fields after treatment [98]. In contrast, surgical outcomes are more variable and generally guarded because manipulation of the optic nerve and its vascular supply carries a substantial risk of further visual deterioration or blindness [1,19,80,124]. Across all treatment strategies, better baseline visual function, shorter symptom duration, and the presence of optic disc swelling rather than established atrophy are associated with a more favourable visual prognosis [117].

## 9. Conclusions

Optic nerve sheath meningiomas are rare, potentially sight-threatening tumors. The diagnosis relies primarily on characteristic clinical features combined with high-resolution MRI and somatostatin receptor-based imaging. Biopsies are generally reserved for atypical or inconclusive cases. Clinically, ONSM presents with slowly progressive, painless visual loss and optic disc changes. Therapeutically, observation remains appropriate in selected patients with good, stable vision, provided that strict, structured follow-up is ensured. Radiotherapy techniques remain the cornerstone of vision-preserving treatment, offering excellent local control and stabilization or improvement of visual function for the majority of patients, with an acceptable and increasingly well characterized toxicity profile [98]. Surgery is considered a secondary treatment in selected cases.

While advances in molecular profiling have generated interest in targeted and immunotherapeutic approaches, their role in ONSM remains largely speculative. Current evidence is derived predominantly from intracranial meningioma studies, and dedicated ONSM-specific data are insufficient to support routine clinical use. Further molecular and clinical investigations are needed to determine whether these strategies may have a role in the management of ONSM.

The long-term prognosis is determined by the visual outcome, requiring timely diagnosis, early intervention in progressive disease, and lifelong surveillance tailored to both tumor behavior and prior treatment.

## Data Availability

No new data were created or analyzed in this study. Data sharing is not applicable to this article.
